# Genome-wide survey and characterization of microsatellites in cashew and design of a web-based microsatellite database: CMDB

**DOI:** 10.3389/fpls.2023.1242025

**Published:** 2023-08-21

**Authors:** Siddanna Savadi, B. M. Muralidhara, V. Venkataravanappa, J. D. Adiga

**Affiliations:** ^1^ ICAR- Directorate of Cashew Research (DCR), Puttur, Karnataka, India; ^2^ ICAR-Indian Institute of Horticultural Research (IIHR), CHES, Madikeri, Karnataka, India

**Keywords:** microsatellite, genome wide, cashew nut, database, validation

## Abstract

The cashew is an edible tree nut crop having a wide range of food and industrial applications. Despite great economic importance, the genome-wide characterization of microsatellites [simple sequence repeats (SSRs)] in cashew is lacking. In this study, we carried out the first comprehensive genome-wide microsatellites/SSRs characterization in cashew and developed polymorphic markers and a web-based microsatellite database. A total of 54526 SSRs were discovered in the cashew genome, with a mean frequency of 153 SSRs/Mb. Among the mined genome-wide SSRs (2-6 bp size motifs), the dinucleotide repeat motifs were dominant (68.98%) followed by the trinucleotides (24.56%). The Class I type of SSRs (≥20 bp) were 45.10%, while Class II repeat motifs (≥12–<20 bp) were 54.89% of the total genomic SSRs discovered here. Further, the AT-rich SSRs occurred more frequently in the cashew genome (84%) compared to the GC-rich SSRs. The validation of the *in silico*-mined genome-wide SSRs by PCR screening in cashew genotypes resulted in the development of 59 polymorphic SSR markers, and the polymorphism information content (PIC) of the polymorphic SSR markers ranged from 0.19 to 0.84. Further, a web-based database, “Cashew Microsatellite Database (CMDB),” was constructed to provide access to the genome-wide SSRs mined in this study as well as transcriptome-based SSRs from our previous study to the research community through a user-friendly searchable interface. Besides, CMDB provides information on experimentally validated SSRs. CMDB permits the retrieval of SSR markers information with the customized search options. Altogether, the genome-wide SSRs characterization, the polymorphic markers and CMDB database developed in this study would serve as valuable marker resources for DNA fingerprinting, germplasm characterization, genetic studies, and molecular breeding in cashew and related *Anacardium* species.

## 1 Introduction

The cashew (*Anacardium occidentale* L.) is an edible tree nut crop grown in more than 30 countries in the tropical and subtropical regions (Sharma et al., 2020). Cashew is an evergreen tree with one or more vegetative and reproductive flushings occurring in an annual cycle (Adiga et al., 2019). It is a diploid species (2n =42) with an andromonoecy breeding system (Aliyu and Awopetu, 2007; Savadi et al., 2020a). It has its origins in North-Eastern Brazil and it was spread to different parts of the world, mainly by the Portuguese during the 16^th^ century as the soil conservation tree (Samal et al., 2003; dos Santos et al., 2019).

Over time, cashew has become an economically important horticulture crop in many developing countries earning huge foreign exchange. Presently, the global raw cashew nut production is over 3.8 million metric tons, and the value of the global cashew nut market is worth nearly US$ 6 billion (INC Statistical Yearbook, 2020–2021). Cashew nut kernels, cashew nut shell liquid (CNSL), kernel oil, and cashew apple (hypocarp or pseudofruit) are utilized in food and numerous other industries (Samal et al., 2003; Talbiersky et al., 2009; Das and Arora, 2017; Sharma et al., 2020). Kernels are used as a dessert nut and in confectioneries such as chocolates, cashew milk, cashew butter, etc. The kernel oil has excellent cooking quality and also widely used in the cosmetic industry due to its nourishing and nurturing effects on skin (Visalakshi et al., 2011). The cashew apple is also edible and is consumed raw or in processed form viz., jam, jelly, syrup, ready-to-serve juice (Akinwale, 2000; Sharma et al., 2020). The cashew apples used to prepare fermented alcoholic beverages (Das and Arora, 2017). CNSL, a reddish-brown oil present in a cashew nutshell, is a multipurpose byproduct of the cashew industry widely used in varnishes, lubricants, synthetic resins, molding compositions, and insulating coatings (Talbiersky et al., 2009; Furtado et al., 2019). Recently, anacardic acid, cardanol, and cardol, the major constituents of CNSL, have acquired great importance in the pharmaceutical industry as they have anticancer and many other great medicinal properties (Ashraf and Rathinasamy, 2018; Shi et al., 2019). Thus, the demand for cashew nuts and their products is increasing throughout the world.

Despite its great economic importance and demand, the genetic improvement of cashew using molecular breeding tools has lagged behind the other important fruit and nut crops due to the limited or lack of genetic and genomic investigations (Savadi et al., 2020a). Molecular markers are important molecular breeding tools extensively used in plant genetics and breeding (Collard and Mackill, 2008). Over the course of time, different types of molecular markers have been developed, which fall into mainly the dominant and co-dominant classes. Co-dominant markers such as Simple Sequence Repeats (SSRs)/Microsatellites and Single Nucleotide Polymorphisms (SNPs) are considered to be more informative and produce consistent results compared to the dominant markers such as RAPD, ISSR and/or AFLP and as a result, the dominant markers are becoming obsolete (Collard and Mackill, 2008; Grover and Sharma, 2016).

To date, in the majority of the genetic studies in cashew, the first generation or dominant markers, viz., RAPD, ISSR, and AFLP markers, have been used (Mneney et al., 2001; Dhanaraj et al., 2003; Archak et al., 2003a; Archak et al., 2003b; Archak et al., 2009; Thimmappaiah et al., 2009; Aliyu, 2012; Jena et al., 2016; Borges et al., 2018; dos Santos et al., 2019; da Costa Gomes et al., 2021) due to the limited availability of co-dominant markers viz., SSR, SNP and InDel (Insertion/Deletion) markers (Croxford et al., 2006; Savadi et al., 2022a; Savadi et al., 2023). Among the co-dominant markers, microsatellite or SSR markers have gained wide popularity and become markers of choice for genetic studies because of their multi-allelism, high abundance in the genome, ease of use, and amenability for high-throughput analysis (Taheri et al., 2018; Savadi et al., 2020b). Presently, only 21 genomic SSR (Croxford et al., 2006) and 36 genic SSR (Savadi et al., 2022a) markers are available in cashew, which is extremely low to represent the entire genome and does not meet the needs of comprehensive genetic research in cashew.

Previously, the development of microsatellite or SSR markers was an expensive and time-consuming process. But the rapid improvements in sequencing technologies and bioinformatics have reduced the cost and time required for the development of a large set of robust markers through genome sequencing and in silico mining of the genome sequences for potential markers (Luo et al., 2021; Wang et al., 2022; Savadi et al., 2023). To date, there have been no studies on the genome-wide characterization of microsatellites or SSRs in cashew. Further, the large set of SSRs mined from the genomic sequences cannot be efficiently utilized by the researchers without a user-friendly analytical search tool. In numerous crops such as *Cucumis melo* (CmMDb: Chaduvula et al., 2015), Sugar beet (SBMDb: Iquebal et al., 2015), Sesame (SisatBase: Dossa et al., 2017), and *Anemone* sp. (Martina et al., 2022), genome-wide SSRs have been discovered and web-based databases are designed for storage and easy accessibility of the large set of genome-wide SSRs to researchers. Recently, the first draft genome of cashew cv. Bhaskara (356 Mb size with 92% BUSCO value) was generated through hybrid assembly of Illumina mate-pair reads and Oxford Nanopore reads and reported by our group (Savadi et al., 2022b). The availability of draft genome sequence prompts the discovery of a large set of SSRs at the genome level and make them easily accessible to researchers.

In the present study, we carried out the comprehensive characterization of genome-wide microsatellites/SSRs for the first time in cashew and designed a user-friendly web-based microsatellite database for easy availability of genome-wide SSR information to cashew researchers. The possibility of *in silico*-discovered SSRs to detect polymorphism in cashew genotypes and cross-amplify in related *Anacardium* species was validated by PCR amplification and fragment separation using a subset of mined SSRs. Thus, microsatellites/SSRs resources generated here would be useful for accelerating genetic studies and crop improvement in cashew and related species.

## 2 Materials and methods

### 2.1 Plant material and DNA extraction

In this study, 32 cashew genotypes (**Table 1**) and two *Anacardium* species, *A. microcarpum* and *A. othonianum*, were used for the validation of polymorphism and cross species amplification of SSRs, respectively. Leaves were harvested from the field-grown plants, and genomic DNA was extracted following the Inglis et al. (2018) method. Initially, finely ground leaf tissues were pre-washed twice with the Sorbitol wash buffer [100 mM tris hydrochloride (Tris-HCl) pH 8.0, 0.35 M Sorbitol, 5 mM ethylenediaminetetraacetic acid (EDTA) pH 8.0, 1% (w/v) polyvinylpyrrolidone (PVP-40)] with 2-Mercaptoethanol (1% v/v) to remove the excessive phenolics. The prewashed samples were then used for DNA extraction with cetyl trimethylammonium bromide (CTAB) buffer [100 mM Tris-HCl pH 8.0, 3 M NaCl, 3% CTAB, 20 mM EDTA, and 1% (w/v) PVP-40]. The integrity of extracted DNA was checked by electrophoresis on a 1% agarose gel containing 0.1 mg/ml of ethidium bromide and quantified using a NanoPhotometer N60 (Implen, Munich, Germany).

**Table 1:** List of genotypes used in this study with important characteristics.

Sl. No	Name	Characteristics
1	NRC-265	Short Flowering duration (<60 days)
2	NRC-346	Bold nut type (7- 12 g) (>7 g)
3	NRC-349	Bold nut type (7- 12 g) (>7 g)
4	NRC-366	Big apple type (>52 g)
5	NRC-383	Big apple type (>52 g)
6	NRC-385	Big apple type (>52 g)
7	NRC-386	Big apple type (>52 g)
8	NRC-458	Small apple type; High shelling % (>28%)
9	NRC-470	High shelling% (>28%)
10	NRC-478	High yielding (6 years cumulative harvests > 18 kg/tree)
11	NRC-20	Long flowering type (> 90 days)
12	NRC-38	High apple to nut ratio (> 12.0)
13	NRC-40	Early Flowering (Nov– Dec)
14	NRC-145	Big apple type (>52 g)
15	NRC-160	High yielding (6 years cumulative harvests > 18 kg/tree)
16	NRC-270	Big apple type (>52 g)
17	NRC-308	High shelling % (>28%) Smooth apple surface
18	NRC-318	High shelling % (>28%)
19	NRC-333	High shelling % (>28%); High kernel weight (> 2.5 g)
20	NRC-335	Low Shelling % (<18%)
21	H-130	Jumbo nut type (> 12 g)
22	Brazil dwarf	Dwarf type (<4 m height)
23	Purple mutant	Purple fruit and shoots
24	NRC-450 (Thaliparamba-1)	Compact type
25	Bhaskara	High yielding (6 years cumulative harvests > 18 kg/tree)
26	NRC-364 (V-7)	Bold nut type (7- 12 g)
27	NRC-188	CNSL free
28	NRC-281	CNSL free
29	NRC-285	CNSL free
30	NRC-473 (Kodippady-1)	Compact type
31	NRC-391	Bold nut type (> 7 g)
32	NRC-152	Lacks ridges on the cashew apple; unique nut shape

### 2.2 Discovery, characterization, and validation of genome-wide SSRs

The whole-genome sequence (356 MB size, 92% BUSCO value) of cashew cv. Bhaskara generated by hybrid assembly of Illumina mate-pair reads and Oxford Nanopore reads at ICAR-DCR (Savadi et al., 2022b) and deposited in the National Centre for Biotechnology Information (NCBI) database (PRJNA766521) was used for the mining of genome-wide microsatellites/SSRs. The PolyMorphPredict software, which permits *in silico* mining of microsatellites and designs primers from genome and transcriptome data, was used to discover and characterize the composition and distributions of genome-wide SSRs (Das et al., 2019). PolyMorphPredict takes input sequence data in Fasta format, mines the SSRs with the MISA tool, and designs primers for the MISA mined SSRs using the Primer3 software with default parameters. The draft genome sequence was mined for SSRs with a minimum repeat number of 6, 5, 5, 4, 3 for di, tri, tetra, penta, and hexanucleotide SSRs, respectively, and a maximum difference of 100 bp between two SSRs. Further, a total of 100 primer pairs were synthesized from Eurofins Genomics, Bengaluru, India, for validation of *in silico* mined SSRs by polymerase chain reaction (PCR) amplification.

### 2.3 PCR amplification of SSRs

The annealing temperatures (Ta) of the synthesized SSR primers were optimized using gradient PCR. The PCR reaction was performed in 15 μl reaction mixtures containing 7.5 μl EmeraldAmp^®^ GT PCR Master Mix (Takara Bio Inc., Japan), 20 pM each of the forward and reverse primers, and 100 ng of genomic DNA in the Veriti™ 96-Well Thermal Cycler (ThermoFisher Scientific USA) and volume makeup was done with Millipore water. The thermal profile conditions used for PCR amplification of SSRs included an initial denaturation step of 3 min at 95°C followed by 35 cycles of 40 s at 95°C, 40 s at primer-specific Ta, 45 s at 72°C and finally, 8 min at 72°C. The PCR products were resolved along with a 100 bp DNA ladder on 3.5% agarose gels containing 0.1 mg/ml of ethidium bromide in 1X TBE (Tris/borate/EDTA) buffer by electrophoresis at 70 V for 3 h. The gels were visualized by exposing them to UV light in the Gel Doc system (Alpha Imager, USA). The primer pairs producing clear and distinct PCR bands in the expected size range on the gel were considered positive amplifications. The primer pairs showing positive amplifications were evaluated for polymorphism detection in 32 cashew genotypes at standardized PCR conditions (**Table 1**), and the primer pairs displaying different-sized bands among the genotypes were considered polymorphic. Further, SSR primer pairs were also tested for cross-species amplification in *A. microcarpum* and *A. othonianum* by PCR screening, and the analysis of PCR products was the same as described above for cashew. The SSR markers producing specific PCR bands in the expected size range were considered cross-transferable to the related *Anacardium* species.

### 2.4 Data analysis

The PCR bands in the gel photos of each SSR primer were scored in the allelic format (band sizes in bp). After scoring the data, genetic diversity, heterozygosity, allele frequencies, allele number, genotype frequency, and polymorphic information content (PIC) values were calculated for each SSR marker using PowerMarker V3 (Liu and Muse, 2005). Dice index-based dissimilarity matrix was calculated and used for clustering analysis of cashew genotypes by the Neighbor-Joining (NJ) method with the DARwin software V6 (Perrier and Jacquemond-Collet, 2006).

### 2.5 Development of the Cashew Microsatellite Database

Cashew Microsatellite Database (CMDB) was designed and implemented as a three-tier architecture website using the tech tools Node js, react js, and MongoDB. Users can create a custom query using the multiple filters available on submission of the search button on the website. An HTTP request is made to the Node JS server where the request is processed, and data will be retrieved from MongoDB and sent back as an HTTP response to the client browser. The basic scheme of CMDB development involved the following steps: i) Genomic and Genic SSR datasets were experimented with, recorded, and consolidated; ii) entities and relationships among the entities in the rational database were created; iii) Database Normalization i.e., organization of data into tables in such a way that the results of using the database are always unambiguous and intended; iv) relationships between the tables were established according to rules designed both to protect the data and to make the database more flexible by eliminating redundancy and inconsistent dependency; and v) design and implementation of a three-tier architecture website using tech tools Node js, react js and MongoDB to make the SSR data accessible to the user.

## 3 Results

### 3.1 Composition and distribution of genome-wide microsatellite/SSRs

A total of 54,526 SSRs were mined from the 356 Mb draft genome sequence of *A. occidentale*, with mean marker density of 153 SSRs per Mb (**Table 2**). However, the primer pairs could be successfully designed for flanking sequences of 47,646 SSRs of the detected SSRs (**Supplementary Table 1**). Analysis of repeat motifs showed that 87.39% of mined SSRs were the perfect type of SSRs, i.e., repeat motifs are continuous without interruption by any nucleotide [e.g., (GC)20], while 12.61% were the imperfect or compound type of SSRs, i.e., SSRs with the stretches of repeat motifs interrupted by nucleotides that are not repeated [e.g., (AT)_12_GC(AT)_8_] (**Table 2**).

**Table 2:** Summary statistics and characteristics of genome-wide SSRs in cashew genome.

Microsatellite Mining	Number	Percentage	Density/Mb
Total number of sequences examined:	3268	–	–
Total size of examined sequences (bp):	356594228	–	–
Total number of identified SSRs:	54526	100	152.77
Number of SSR containing sequences:	2572	78.70	–
Number of sequences containing ≥1 SSR:	2132	65.23	–
Di-nucleotide SSRs	37614	68.98	105.66
Tri-nucleotide SSRs	13395	24.57	37.63
Tetra-nucleotide SSRs	2560	4.70	7.19
Penta-nucleotide SSRs	523	0.96	1.47
Hexa-nucleotide SSRs	434	0.80	1.22
Compound SSRs	6880	12.62	19.33

**Figure 1:**
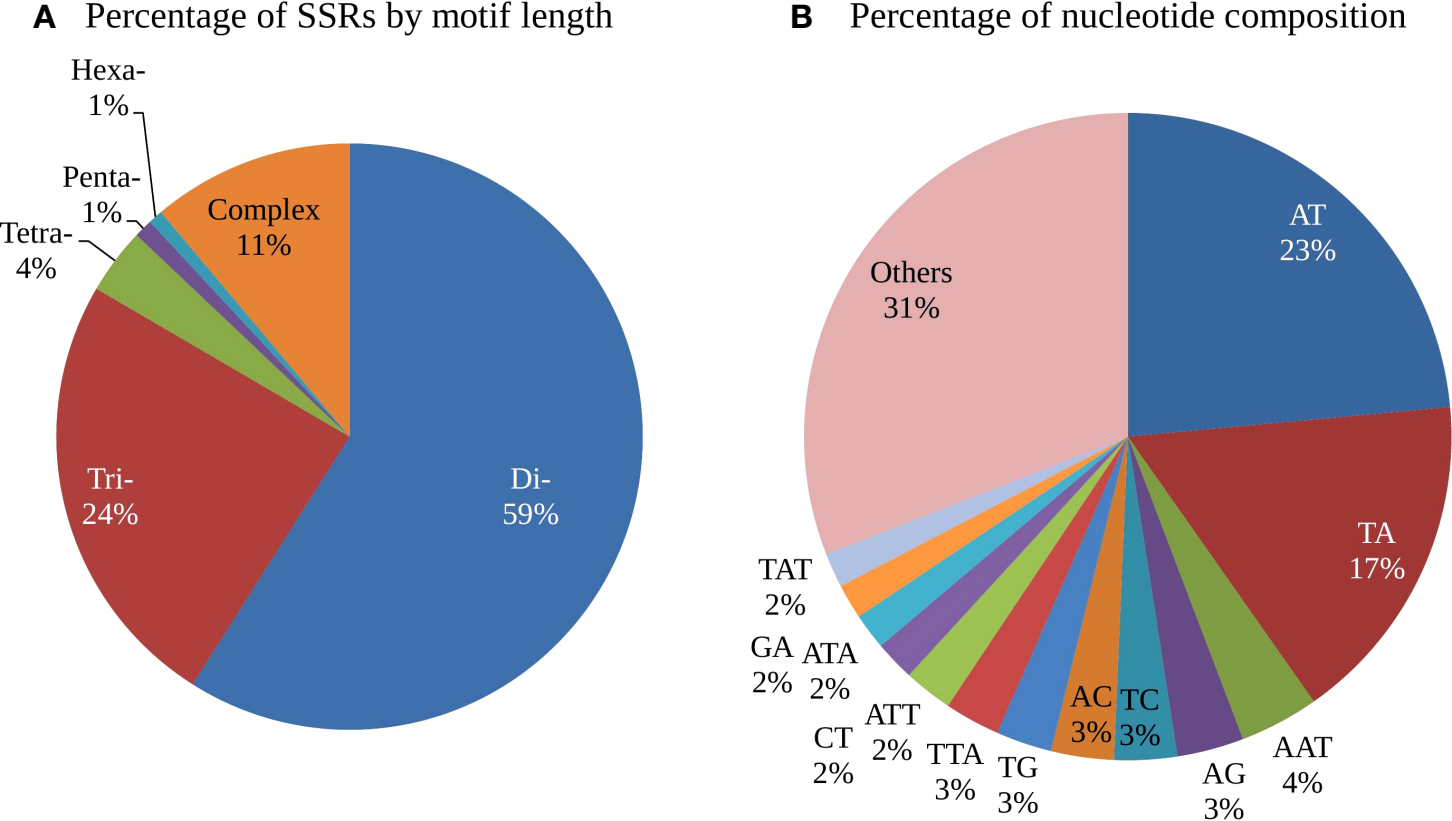
Frequency distribution of different types of SSR repeats in the Cashew genome **(A)** Frequency of motif types by unit length (K-mers) **(B)** Frequency of repeat motifs by nucleotide composition.

Analysis of the distribution of the five classes of perfect SSRs in the cashew genome revealed that the dinucleotide repeat types were most abundant, comprising 68.98%, followed by the trinucleotide repeat motifs (24.57%), the compound SSR repeats (12.62%), the tetra-nucleotide repeat motifs (4.70%), the pentanucleotide repeat motifs (0.96%), and the hexanucleotide repeat motifs (0.80%) (**Table 2**).The frequency distribution of different repeat motifs in the draft genome of cashew is presented in **Figure 1A**. The nucleotide composition of the identified SSRs showed that 84% were composed of A and/or T nucleotides, while 16% were composed of G and/or C nucleotides. The most dominant repeat sequences were AT (23.54%), followed by TA (16.71%) and AAT (4%) (**Figure 1B**). The detailed frequency distribution of repeat motifs and the repeat numbers in the di- and tri-nucleotide SSRs, which are dominant in the genome-wide SSRs, is presented in **Table 3**. In the di-nucleotide SSRs, AT/AT repeat motifs were most abundant (65.79%) and CG/CG repeats were the least abundant repeat motifs (0.30), and the remaining two types, AC/GT and AG/CT, were 17.38% and 16.49%, respectively. In the trinucleotide SSRs, AAT/ATT repeat motifs were most abundant (55.37%), followed by AAG/CTT with a frequency of 22.46%, and ATC/ATG repeat motifs were 8.94%, ACC/GGT motifs were 3.81%, AAC/GTT motifs were 3.10%, AGC/CTG motifs were 2.31%, AGG/CCT motifs were 2.61%, and other motifs (ACG/CGT, ACT/AGT, and CGG/CGG) together were 1.39%.

**Table 3:** Frequencies of different repeat motifs in di- and tri-nucleotide SSRs in cashew genome.

Repeat Motif	Repeat number								
	5	6	7	8	9	10	> 10	Total	%
AC/GT	–	1829	1064	727	660	454	1805	6539	17.38
AG/CT	–	2035	967	611	570	418	1603	6204	16.49
AT/AT	–	5776	3701	2964	2279	1782	8245	24747	65.79
CG/CG	–	91	20	11	2	–	0	124	0.33
Total	–	9731	5752	4313	3511	2654	11653	37614	–

AAC/GTT	233	77	56	26	10	7	6	415	3.10
AAG/CTT	1257	661	389	241	172	101	188	3009	22.46
AAT/ATT	3291	1652	972	593	336	220	353	7417	55.37
ACC/GGT	266	123	56	37	13	8	8	511	3.81
ACG/CGT	29	12	8	2	1	1	0	53	0.40
ACT/AGT	36	18	10	5	5	1	6	81	0.60
AGC/CTG	210	68	24	8	–	–	0	310	2.31
AGG/CCT	149	95	34	28	21	11	12	350	2.61
ATC/ATG	631	309	124	58	33	18	24	1197	8.94
CCG/CGG	39	7	4		1	1	0	52	0.39
Total	6141	3022	1677	998	592	368	597	13395	–

Based on the size of repeats motif, the mined SSRs were categorized into two classes, viz., Class I (hypervariable) SSRs, the SSRs with repeat motif size ≥20 bp, and Class II (variable) SSRs, the SSRs with repeat motif sizes of ≥12 and <20 bp. The frequency of Class I genomic SSRs was 45.10%, while that of Class II types was 54.89%. The Class I SSRs were dominated by the dinucleotide (46.71%) and the compound SSRs (24.66%), while the Class II SSRs were dominated by the dinucleotide (69.01%) and the tri-nucleotide SSRs (30.92%).

### 3.2 Polymorphism, genetic diversity, and transferability of genomic SSR markers

**Figure 2:**
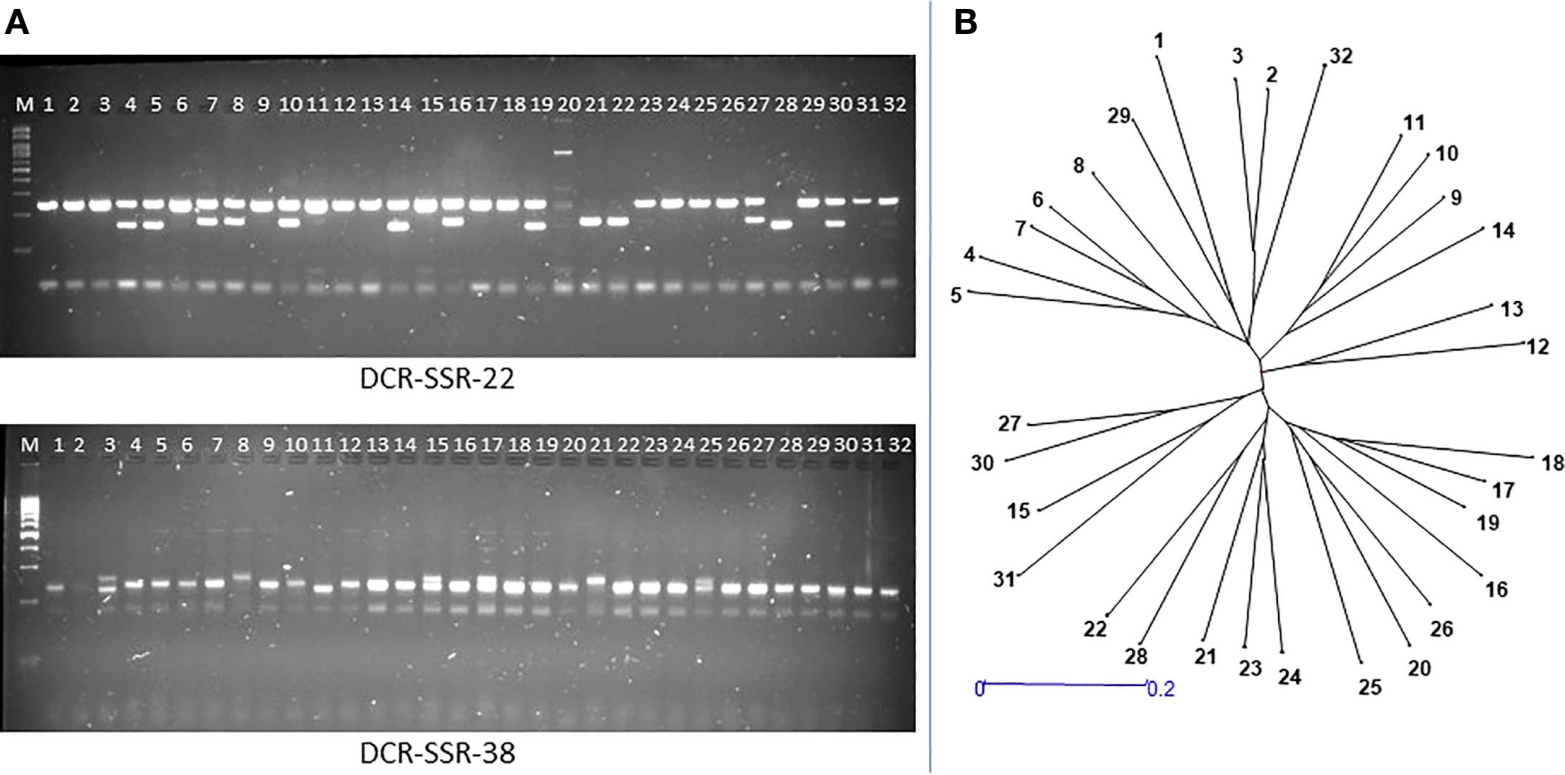
Validation of genome-wide SSRs for amplification and polymorphism in 32 cashew genotypes: **(A)** Gel pictures showing polymorphism detection by the DCR SSR-22 and DCR SSR-38 markers in 32 cashew genotypes; **(B)** Neighbour-Joining dendrogram showing genetic relationships among 32 cashew accessions collected from different geographic regions. The dendrogram is constructed based on Nei’s (D) genetic distance coefficient.

We validated the mined genomic SSR markers by synthesizing 100 primer pairs and testing them for PCR amplification in *A. occidentale.* All the tested SSR primer pairs were successfully amplified in *A. occidentale*, indicating 100% accuracy in primer design. Further, fifty nine of 100 primer pairs screened in 32 germplasm accessions showed polymorphism (**Table 4**). The 59 polymorphic markers detected 294 alleles in 32 accessions. The number of alleles per SSR locus varied from 2 to 15, with a mean of 4.98 alleles per SSR locus (**Table 4**). PIC values of the assayed SSRs ranged from 0.19 to 0.84, with a mean of 0.59 (**Table 4**). Polymorphic SSR markers were grouped into three classes based on the PIC values. Of the 59 SSR markers developed, 47 were highly polymorphic (PIC value ≥0.50), 9 were moderately polymorphic (PIC value between 0.25-0.50), and 3 markers were least polymorphic (PIC value <0.25) (**Table 4**). Further, 39 of the polymorphic SSRs were of di-nucleotide type, 8 were of trinucleotide type, and 12 were compound SSR repeats (3 perfect and 9 imperfect type compound SSRs). The wide range in amplicon size difference (~150–250 bp) was observed with the marker DCR SSR-22 amplifying a compound motif: (GT)6ct(GA)15 (**Figure 2A**).

**Table 4:** Characteristics of the 59 novel polymorphic SSR markers developed in this study by scanning of the whole genome sequence of cashew for SSRs and validation.

Sl No.	Marker	SSR primer (F&R)	Ta (°C)	Repeat motif	Allele size range bp	Allele No	PIC
1.	DCR SSR-1	GAAACACCTGTTCCACACGC	54	(AT)15	~260-325	5	0.66
		CCTTGACCTCGTGCCAATCT					
2.	DCR SSR-3	AGTTTGCCGAAGCTCTCAACT	54	(TA)7	~275-320	4	0.69
		GCGGAAGTAGTCTTTTTGCCA					
3.	DCR SSR-6	ATACGTCCAACAAACGCCCT	53	(AT)10	~225-275	3	0.48
		AGCGAACGATGTTGTTTTGGT					
4.	DCR SSR-8	TGCTGCACAGAGAGACTTGG	54	(TTC)5	~200-225	4	0.64
		AGGAAGATTTGCCTGCAGCT					
5.	DCR SSR-9	TGGCTATTTCCTTGGGCAGG	53	(CT)7	~225-275	5	0.56
		TTTTTCCTCTCAGCCGTCCC					
6.	DCR SSR-10	GGTTGTTGAGTAGCAGGGGT	56	(TA)29	~250-300	4	0.61
		ACAACTTGCAATTGTGGT					
7.	DCR SSR-11	GCGTACACACACACACACAC	54	(AC)9	~275-300	3	0.51
		GCGAATGGGAAGTTGCCAAG					
8.	DCR SSR-12	TTTCCACGCCTACCACAGTC	54	(TA)25	~250-300	4	0.52
		AGAGGGGAAAAGTGCAAGCA					
9.	DCR SSR-13	GAGGTTGATCCACAGCAGCT	55	(TTG)8	~250-300	4	0.67
		GCATTGCAGGCACAAGAACA					
10.	DCR SSR-14	GATCACACGCACGATGAAGC	56	(AT)6	~230-300	5	0.6
		CAAGCCCCCTTTTTCTGTGC					
11.	DCR SSR-15	CGCGATGGGAATTCTACGGA	54	(AT)20	~260-300	3	0.58
		CCCACACAGCGATCTCAGTT					
12.	DCR SSR-17	GGTGGTATGTTGGAAGCCCA	52	(AT)16	~240-275	6	0.67
		TGGGAGCTAACCTAGAGCACA					
13.	DCR SSR-20	ATCGCCACCTACCCAACAAG	52	(AATA)5(AT)22	~250-275	15	0.82
		GCAATGCAAAGTATGAGGGTGG					
14.	DCR SSR-21	TGGATTTGAGAGGGTTCAAGGG	56	(AT)7gtatatgtatgtgtgtatatatatg(TA)11	~175-240	3	0.59
		ACACACGAGTCTGTGTCCAA					
15.	DCR SSR-22	CCGTGTGTGTGTGTCTGAGA	54	(GT)6ct(GA)15	~150-250	5	0.66
		GCTGAGGCATCTCTTTGGGT					
16.	DCR SSR-23	AGTCATCGTCGCTGATTACG	54	(AT)35	~175-250	4	0.57
		ACCTGCTGTGTTGATCAGACT					
17.	DCR SSR-28	AGATGTGTGTGGGCTTCAGG	54	(AC)9ag(AT)18	~250-300	4	0.64
		TCCGCACTCTTCAGCTTGTT					
18.	DCR SSR-29	TGGTTGGATTTCCCCTTGGA	54	(AT)39	~175-250	4	0.57
		ATTAACTGAACGCGAGCCCA					
19.	DCR SSR-30	ACACTTCCCATGAACAAGGACT		(AT)15	~240-300	4	0.63
		CATTAGCTCGAGGCCTGACA	54				
20.	DCR SSR-32	CTGGCTAACGGGAGGTTTGT		(GT)6(AT)10	~245-290	6	0.76
		GAGAGAGGGAGAAGGGGAGG					
21.	DCR SSR-33	ACCAATCCCACCAGCAACAA	54	(TG)10tctgtgtctctgtg(TGTGTC)5 tgtgtgtctgtgc(AT)7acaca cacacatatatgtgtgtctgtgtgtctgtgc(AT) 7acacacacatata(TG)20 tctgtgc(AT)7acacacata(TG)6tctgtgc (AT)11acacacacac(GT)9	~200-250	6	0.67
		GCACAGACACACACACACAC					
22.	DCR SSR-34	CTCTACCCACTCACCGAGGA	54	(AT)21	~220-275	5	0.53
		AGCACGTTCCACAAGGTTCA					
23.	DCR SSR-35	TGGAGGTGTTTGGGATGCAA	54	(AT)16	~250-300	6	0.7
		TCCCCATTTGTGGTTGTGCA					
24.	DCR SSR-36	GTCTGATCAGCACAGCAGGT	54	(AT)6	~250-275	3	0.44
		CCCTCAAAACCCAAGCAAGC					
25.	DCR SSR-37	GGGTGGGGTGAGTTTCCATT	54	(TAT)8	~230-250	5	0.6
		GGCCAACCCAGCTTGAAATG					
26.	DCR SSR-38	GGCAACACGTCACCTGGATA	54	(AT)29	~200-250	11	0.84
		TTAAGAATGCCTGGGCCACA					
27.	DCR SSR-39	AATAAGAGCACCTCGGCAGC	54	(TA)14	~250-275	3	0.29
		GCACGTTGCACATGTTTTCG					
28.	DCR SSR-41	TGTCTACACCTGTTTCTCCGT	56	(TA)9	~250-300	6	0.56
		GGCAAGTAGTAGCTCCACCC					
29.	DCR SSR-42	CAAGAGGCCCCAAAAACAGC	54	(AT)17	~250-325	6	0.72
		GCCTGCCACCCTCACAATAT					
30.	DCR SSR-43	TGTCCAGGGAGAGTGACTGT	52	(AT)22	~250-300	6	0.7
		TTTCAGCTGGCATGCCCATA					
31.	DCR SSR-44	TTTTGGGTTGGCAATGGCTG	54	(AC)7aa(AT)25(AC)9aa(AT)28	~225-250	5	0.66
		CTCATCGAGGTTGGTTGCCT					
32.	DCR SSR-45	CCCCTGCAATTTTCCACGTG	54	(AT)16	~250-275	6	0.63
		AAACCCTCAGAGCTGATGCC					
33.	DCR SSR-46	GTTGGAACAGGCGACCTACA	54	(AT)46	~190-250	4	0.48
		ACCATTTCGAACTGGAGCCA					
34.	DCR SSR-47	ACCCACAGCTAGCCCAAATC	54	(AT)11	~225-275	5	0.71
		GGCAAGCCTAGGCCTACAAA					
35.	DCR SSR-49	GCGGGAAAACATGTGGTGTG	54	(TA)10aggttttccc(AT)10	~200–225	4	0.63
		CATGTTGTGGCTTGCATGCT					
36.	DCR SSR-51	GGGGTGAGTAGTTGGCCATC	54	(TAA)7tgcaatacctgattgaattcagaagctg Aatggttgaattggtcccacgggatgggggtgagta gttggccatc(ATA)6	~225-250	5	0.71
		AGGAGCTGTACGCAAAACCA					
37.	DCR SSR-52	GCTTGAGTTTGGCTTGGCTT	54	(TA)19	~200-225	2	0.38
		TCCACACAAGGCCACAAGTT					
38.	DCR SSR-53	TGGAGTTACCCACCTGTACCA	54	(TA)6	~225-275	4	0.62
		GGCTGTGAAGAAGTGTTTCGC					
39.	DCR SSR-56	GGCTTGAGTGCTGAATCCCT	54	(TA)34	~225-250	2	0.3
		TCATAAACTTGCCCTTGGGCA					
40.	DCR SSR-58	TGCTCCTGCCTTTGTGCTAA	54	(AC)7	~200-225	4	0.64
		ACACGAAAACTTCAACGTGGG					
41.	DCR SSR-59	ACCCAGTTTAACAGGCTGAA	54	(TAA)5	~200-250	4	0.7
		TTTTCCTCCGCTTCTCGCAT					
42.	DCR SSR-60	GGGAATGGTGGGGGTAAAGG	55	(TGG)5c(GGA)5ggtggaggtggtc gtactaatggtggagggtatggtcatg gaagtggtttcggggcaggaggcggagcaggaa (GTG)6(GAG)5	~225-250	5	0.56
		TCTCATCAGAGCCACCTCCA					
43.	DCR SSR-62	TCCTTGCTGGCGACTTTGAT	54	(GT)12(AT)8	~175-200	4	0.58
		TTCGTCCCCTTCCTCTTCCT					
44.	DCR SSR-63	TGGATTCTCCCTTCCCCCTT	54	(TC)16	~225-250	2	0.37
		AATAGGCCAGGGAGGTGAGA					
45.	DCR SSR-67	TGATAGGCCGAATCAGCGAC	54	(CCA)7	~200-225	4	0.6
		ACTATTGTTCTCTGGCGGCC					
46.	DCR SSR-68	GGCAGTGTCTGGTAGGTCAC	54	(AG)9acggtacccaaacatttactattagtgctcc ttatcatggctttgaaatatgg(AT)9	~225-250	2	0.19
		TTGATCAAGAGGCCAGTCCG					
47.	DCR SSR-69	CAAGCAGCAAACAAGGGCAT	54	(TA)7	~200-250	3	0.46
		AACACAGCAGGTACCTTCGG					
48.	DCR SSR-70	CGGTAGGTTTTAGGGCTCCG	54	(AG)7	~200-250	2	0.36
		AACTCATGGGGCACTGTCTG					
49.	DCR SSR-71	GGAAATCCGGGAAAGGGTCA	54	(TA)10	~200-225	2	0.19
		GGCCAGCCAACCTAATGACT					
50.	DCR SSR-72	GTTTGGTAGAGGGATCCTGCA	54	(TA)20	~175-200	2	0.19
		GAAGGTGTTACGGTGGCTCA					
51.	DCR SSR-73	CACAGAGGTGCATGATTTGGTG	56	(AT)8	~210-240	6	0.65
		GGTGCTTTCAACGATCATGGA					
52.	DCR SSR-74	CACCTCCCTCTACAAACGCA	56	(AT)8	~190-220	9	0.81
		AGAGGCTCGGGTAAGTGAGT					
53.	DCR SSR-75	AACCAACAAGTCGGGTGGTT	56	(AAG)8	~170-200	9	0.77
		GTCCCCTCCCAGGTAAGGAT					
54.	DCR SSR-76	GGACTCATATGTGGTGTCGCT	56	(AT)11	~190-220	6	0.71
		ATTCGATCTGTGCTGGCTCC					
55.	DCR SSR-77	AACTGCAGGTCTCTGGGAGA	56	(ATA)10	~100-130	6	0.54
		CTCGCCCTCTCTTGTCAACA					
56.	DCR SSR-78	ACCCTTGCGTTATGTGCACT	56	(AG)12	~240-260	7	0.76
		GCCAACACCAGCACTTGAAA					
57.	DCR SSR-79	CATGGGAGCATCTGGACCAG	56	(TA)6	~185-210	10	0.82
		AGTCTTGGCTCTGTTGTGCA					
58.	DCR SSR-80	TGGAAAGGCTGGAGAAGAGA	56	(TTA)11	~275-290	12	0.8
		GGTTGAATCAAGGTTCGTTGCA					
59.	DCR SSR-81	TAGAAGGGCTTGGGAGTGGA	56	(AT)13	~250-275	6	0.75
		ATCCACTCCCAGACCCTAGG					

The dendrogram analysis of 32 genotypes using the 59 polymorphic SSR markers classified the assayed genotypes into three major clusters (**Figure 2B**). The first cluster consisted of 16 genotypes; the second cluster consisted of 14 genotypes; and the third cluster consisted of two genotypes. Pairwise dissimilarity was a maximum of 0.85 between NRC-335 and NRC-265, while a minimum of 0.33 was observed between NRC-385 and NRC-386. NRC-385 and NRC-386 are the two genotypes originating from a common parent, as depicted in the dendrogram analysis. Further, the NRC-335 and NRC-265 are from two geographically distinct regions, i.e., the NRC-335 is from the West Coast region of India, while the NRC-265 is from the East Coast region of India.

So far, there are no species-specific genetic markers designed for less studied species in the *Anacardium* genus. Cross-species transferability of cashew SSRs can be an alternative source of molecular markers for less studied *Anacardium* species. Testing of cross-species PCR amplification of the newly designed SSR primers in the two *Anacardium* species, viz., *A. microcarpum* and *A. othonianum*, showed that 91% of the tested primers were successful in PCR amplification (**Supplementary Table 2**), suggesting a high rate of transferability of cashew genomic SSRs in the *Anacardium* genus.

### 3.3 CMDB: microsatellite database for cashew

**Figure 3:**
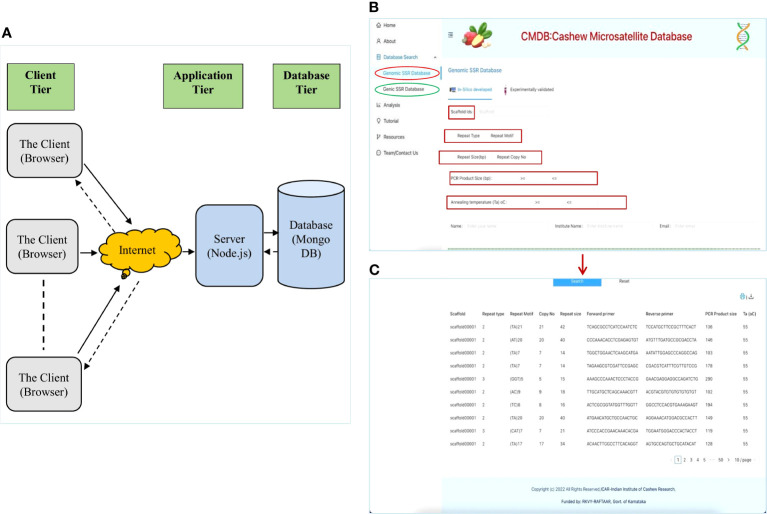
The interface and searching of the Cashew Microsatellite Database (CMDB) for SSRs: **(A)** The three-tier architecture of CMDB, **(B)** The database search page displaying different SSR search parameters; and **(C)** The database search results displaying the details of SSRs.

The Cashew Microsatellite Database (CMDB) is an online relational database that stores the microsatellite repeats information mined from the recently sequenced cashew genome (Savadi et al., 2022b) and the shoot transcriptome (Savadi et al., 2022a), as well as the experimentally validated SSR markers. CMBD is available at https://www.cashewmicrosatellitesdatabase.in/. CMBD is an interactive database that has been implemented as a 3-tier application architecture where we have a Client tier, an Application or Server tier, and a Database tier, as shown in **Figure 3A**. CMDB has a user-friendly interface developed using React js and a server designed and implemented using Node js that connects to MongoDB, where all the genomic and genic SSR data is stored. Users can access this responsive website using any browser on a desktop or mobile device connected to the internet. User-need-based customized queries can be generated from the web interface and allow users to search the Cashew microsatellite database in MongoDB.

CMDB can be searched to extract genomic as well as genic microsatellites based on motif type (di, tri, tetra, penta, and hexa), repeat motif, copy number, repeat size, expected PCR product size and primer pair annealing temperatures (Ta) (**Figure 3B**). The microsatellites can be searched based on the choice of scaffolds/transcripts, where more than one scaffold/transcript can be selected using the dropdown option (**Figure 3B**) the results of the database search displays details of SSRs including the primer pairs for the displayed SSRs (**Figure 3C**). This is a novel approach and is helpful for breeders and biotechnologists to easily extract microsatellites based on their needs.

## 4 Discussion

Though the integration of molecular markers in breeding programs and genetic studies has substantially enhanced the speed and accuracy of crop improvement in important fruit and nut crops, molecular breeding and genetic analysis in cashew have lagged behind due to the scarcity of informative markers. Microsatellites or SSRs are highly informative markers and are widely used in genetic analyses and breeding of crops, including trees. In *A. occidentale*, very limited SSR markers are available for comprehensive genetic studies and molecular breeding (Croxford et al., 2006; Savadi et al., 2022a).

The NGS technologies allow rapid large-scale sequencings at a lesser cost, which permits discovery and development of SSR markers for less studied crops (Khodaeiaminjan et al., 2018; Taheri et al., 2018; Luo et al., 2021; Wang et al., 2022). The genome sequences generated using the NGS technology have been used to discover genome-wide SSRs and develop SSR markers in various tree species, such as pistachio (Ziya Motalebipour et al., 2016), hazelnut (Öztürk et al., 2018), avocado (Ge et al., 2019), fruit and forest species (Song et al., 2021), and *Grevillea* sp. (Dabral et al., 2021). Efficient utilization of the large set of SSRs mined through genome scanning is possible with a user-friendly web tool to search the SSR information from a database. So far, genome-wide SSRs discovery and development of a database for storage and retrieval of the discovered SSRs have not been reported in cashew. The present study, for the first time, reports the discovery and characterization of genome-wide SSRs and the development of a microsatellite database (CMDB) for cashew.

In this study, a total of 54526 SSRs were discovered from the cashew genome, with a mean density of 153 SSRs/Mb. The density of markers found in this study was higher than that reported in apple (40.8 SSRs/Mb, 485 Mb) (Zhang et al., 2021b), Chinese spring wheat (36.68 SSRs/Mb, 9.93 Gb) (Han et al., 2015), and *Matthiola incana* (23.25 SSRs/Mb, 1977.48 Mb) (Tan et al., 2023), while it was less than mango (418.17 SSRs/Mb, 253.6 Mb) (Ravishankar et al., 2015), *Prunus mume* (794 SSRs/Mb, 237 Mb) (Sun et al., 2013), and Pomegranate (527.97 SSRs/Mb, 296 Mb) (Patil et al., 2020), suggesting that generally, the density of SSRs decrease with an increase in the genome size. Further, the frequency of perfect SSRs (87.39%) was much higher than the frequency of imperfect SSRs (12.61%) in the discovered genome-wide SSRs and is consistent with similar results in eggplant (Portis et al., 2018), *Anemone coronaria* (Martina et al., 2022), and *Aristotelia chilensis* (Bastías et al., 2016). In the perfect SSRs, dinucleotide repeat types were most dominant (68.98%), followed by trinucleotide repeat motifs (24.56%), which are consistent with similar results in other plant species investigations (Parida et al., 2015; Vieira et al., 2016; Ziya Motalebipour et al., 2016; Wang et al., 2018).

The most common dinucleotide was AT/AT repeat motifs (65.79%), while CG/CG repeats were the least abundant (0.30). This result is in agreement with previous findings that AT-rich SSRs are predominant in dicots, viz., apple (Zhang et al., 2012a), sweet orange (Biswas et al., 2014), and *Cucumis sativus* (Cavagnaro et al., 2010), while GC-rich dinucleotide repeats are dominant in monocots (Sonah et al., 2011; Qin et al., 2015). These differences in the SSR nucleotide among the dicots and monocots could be partially explained based on the relative nucleotide composition of the genomes. The average GC content of dicot genomes (34.6%) is lower than that of monocot genomes (43.7%) (Cavagnaro et al., 2010), and it is observed that the frequency of AT and TA in the genomes increased with the evolution of the plant kingdom (Qin et al., 2013).

In this study, Class I type of SSRs (≥20 bp repeat motif) were 45.10%, while Class II types (≥12 and <20 bp repeat motif) were 54.89%. The frequencies of two classes of SSRs are in agreement with other studies in plants (Parida et al., 2015; Wang et al., 2018; Patil et al., 2021). Class I SSRs are observed to be highly polymorphic compared to Class II SSRs (Parida et al., 2015; Vieira et al., 2016; Patil et al., 2021) because shorter SSR sequences tend to have lower mutation rates (Vieira et al., 2016).

The validation of genome-wide SSRs was performed by the synthesis and screening of 100 randomly selected SSR primer pairs in cashew genotypes. Fifty nine of the 100 SSR primers screened in 32 germplasm accessions showed polymorphism. To date, 21 genomic and 36 genic SSRs have been reported in cashew (Croxford et al., 2006; Savadi et al., 2022a). Therefore, this study not only provides genome-wide SSR information but also experimentally validated polymorphic SSR markers for cashew. Further, SSR markers grouped based on the PIC values showed that 47 newly developed SSR markers were highly polymorphic (PIC value ≥0.5), 9 were moderately polymorphic (PIC value between 0.25 and 0.50), and 3 markers were least polymorphic (PIC value <0.25) according to the Botstein et al. (1980) classification of polymorphic markers. Furthermore, the grouping of polymorphic SSRs based on the five classes of perfect SSRs and compound SSRs showed that the dinucleotides were dominant. The higher tendency of dinucleotide SSRs to be polymorphic is consistent with other studies on genomic SSRs in apples (Silfverberg-Dilworth et al., 2006), peanuts (Zhao et al., 2012a), watermelon (Zhao et al., 2012b), and black pepper (Negi et al., 2022). However, in a previous study where a repeat-rich genomic library screening method was used to generate SSR markers, the compound SSRs were found to be more polymorphic in cashew (Croxford et al., 2006). This difference in the polymorphic SSRs motif size could be due to the biases caused by the repeat probe sequences (AC15, AG15, AAC8, AAG8, AAT8, ACC8, AGG8, ATC8, AAAC6, AAAG6, and ACAT6) used to screen the genomic libraries for SSRs. The genetic diversity analysis using the newly developed SSRs clustered the 32 genotypes into three major clusters. Further, the pairwise dissimilarity index revealed that the maximum distinction was observed between the accessions viz., NRC-335 and NRC-265 collected from different geographic regions, the West Coast and the East Coast of India, respectively, while the minimum pairwise dissimilarity index was observed between the two genotypes, viz., NRC-385 and NRC-386, which shared one of the parents, indicating that the newly developed genomic SSRs have high discriminating power and present a powerful molecular tool for investigating genetic diversity and genetic relationships in the cashew genotypes.

To our knowledge, there is no distinct set of microsatellites or SSR markers developed for other species in the *Anacardium* genus. It has been demonstrated that SSR markers have a high potential for cross-species amplification/transferability in related species of the same genus. Transferability of markers is considered a cost-effective approach for developing genetic markers for species lacking genomic resources (Ellis and Burke, 2007; Vieira et al., 2016). In this study, 91% of the SSR primer pairs showed cross-species amplifications in the two wild relatives of cashew, viz., *A. microcarpum* and *A. othonianum*. This transferability rate was comparable with the results observed in previous studies using genomic SSRs in cashew (Croxford et al., 2006; Soares et al., 2013), but lower than the transferability with the genic SSRs (Savadi et al., 2022a). In *A. humile*, 85% of the 14 tested cashew SSRs amplified (Soares et al., 2013); in *A. microcarpum*, *A. pumilum*, and *A. nanum*, 92% of the 12 cashew SSRs amplified (Croxford et al., 2006); and in *A. microcarpum* and *A. othonianum*, 100% of 54 transcriptome-based SSRs amplified (Savadi et al., 2022a). The relatively lower rate of transferability of genomic SSRs compared to genic SSRs could be attributed to the higher conservation of genic sequences compared to sequences from the anonymous regions of the genomes (Ellis and Burke, 2007; Jiang et al., 2020). Thus, we contemplate that the SSR markers developed in this study can be a potential marker repository for not only cashew but also the related *Anacardium* species and could be employed for macro-syntenic comparisons, germplasm characterizations, genetic mapping, molecular breeding involving interspecies hybridizations, etc.

With the mining of genome-wide microsatellites/SSRs information, there is a need to develop a user-friendly web tool for easy access and efficient utilization of the mined SSRs in genetic studies and crop improvement. Several databases of genome-wide SSRs have been designed in different crop plants (Arora et al., 2013; Dossa et al., 2017; Jasrotia et al., 2019; Martina et al., 2022). However, in cashew, genome-wide SSRs information is not available. In this study, genome-wide SSR information generated in this study as well as in our previous study from transcriptome data (Savadi et al., 2022a) was integrated into CMDB, which permits the extraction of information related to both genomic and genic SSRs. Further, CMDB also provides the experimentally validated SSR markers in the cashew. Most of the microsatellite databases developed in other crops (Arora et al., 2013; Dossa et al., 2017; Jasrotia et al., 2019; Martina et al., 2022) provide only the *in silico* mined SSRs information of either the genomic SSRs or transcriptomic SSRs but not combined information like the CMDB. Thus, CMDB is the first comprehensive microsatellite database for cashew, and it will be of great use to cashew researchers, particularly the breeders, to develop novel markers from *in silico* mined SSRs and to directly use the experimentally validated markers in the research programs.

## 5 Conclusion

The limited availability of microsatellite/SSR markers in cashew has hindered genetic studies and crop improvement. In the current study, we mined and characterized genome-wide SSRs in the cashew genome and developed a cashew microsatellite database (CMDB), a comprehensive repository of microsatellites, which provides accessibility to genome-wide and transcriptome based SSRs information as well as the experimentally validated SSR markers to researchers and breeders. The large set of genome-wide SSRs and their free public accessibility will permit the development of a large set of new SSR markers for cashew, which are currently very scarce. Besides, we developed 59 highly informative SSR markers that are the first set of genomic SSRs developed in cashew through *in silico* mining of the cashew genome. Thus, the knowledge of genome-wide SSRs distribution, the development of novel SSR markers, the cross-species transferable SSRs, and the comprehensive microsatellite database would significantly accelerate genetic studies and crop improvement in cashew and related *Anacardium* species.

## Data availability statement

The original contributions presented in the study are included in the article/**Supplementary Material**. Further inquiries can be directed to the corresponding author.

## Author contributions

SS conceived the idea, carried out the *in silico* analysis and experiments and development of database and wrote the manuscript. BM contributed in the experimentation and development of database. VV contributed towards sampling and experimentation. JA contributed to manuscript writing and proof editing and development of database.
