# Supplementary material for: Genome-wide survey and characterization of microsatellites in cashew and design of a web-based microsatellite database: CMDB
Source: Front Plant Sci. 2023 Aug 21;14:1242025. doi: 10.3389/fpls.2023.1242025 (PMC10475544; doi:10.3389/fpls.2023.1242025)
Supplement: Supplementary file 2 [file Table_2.docx]

Supplementary Material

Genome-wide survey and characterization of microsatellites in cashew and design of a web-based microsatellite database: CMDB

Siddanna Savadi*^#1^, Muralidhara B. M^2^., V. Venkataravanappa ^2^., J.D. Adiga^1^

^1^ICAR- Directorate of Cashew Research (DCR), Puttur- 574 202, D.K., Karnataka, India

^2^ICAR-Indian Institute of Horticultural Research (IIHR), CHES, Chettalli, Kodagu, Karnataka, India

*** Correspondence:** Corresponding Author: [siddannasavadi@gmail.com](mailto:siddannasavadi@gmail.com)

**Supplementary Table 2** Details of 100 cashew SSR primers designed for validation of the mined genome wide SSRs for PCR amplification and polymorphism detection in A. occidentale, and cross species PCR amplification in A. microcarpum and A. othonianum.

| **Primer name** | **Forward Primer** | **Reverse Primer** | **Product**  **size (bp)** | **Ta (^o^C)** |
| --- | --- | --- | --- | --- |
| DCR SSR-1 | GAAACACCTGTTCCACACGC | CCTTGACCTCGTGCCAATCT | 260-325 | 54 |
| DCR SSR-2 | ACGGCAAAATGACACTCCTT | CGTGAAGCAAGAGAATTGAGCA | 250 | 54 |
| DCR SSR-3 | AGTTTGCCGAAGCTCTCAACT | GCGGAAGTAGTCTTTTTGCCA | 275-320 | 53 |
| DCR SSR-4 | CTACTCGTGCACGACCATGT | CGAGAAGCACACAGTCTCGT | NCA | 54 |
| DCR SSR-5 | GCTTTCGAAGTGGGGCAATC | GGGTCCAAAATGGGGTGTCT | 190 | 54 |
| DCR SSR-6 | ATACGTCCAACAAACGCCCT | AGCGAACGATGTTGTTTTGGT | 225-275 | 53 |
| DCR SSR-7 | TTACCTCTCGACATGGCTGC | GAAGCGCGGGAAATGGATTC | NCA | 54 |
| DCR SSR-8 | TGCTGCACAGAGAGACTTGG | AGGAAGATTTGCCTGCAGCT | 200-225 | 54 |
| DCR SSR-9 | TGGCTATTTCCTTGGGCAGG | TTTTTCCTCTCAGCCGTCCC | 225-275 | 53 |
| DCR SSR-10 | GGTTGTTGAGTAGCAGGGGT | ACAACTTGCAATTGTGGT | 250-300 | 56 |
| DCR SSR-11 | GCGTACACACACACACACAC | GCGAATGGGAAGTTGCCAAG | 275-300 | 54 |
| DCR SSR-12 | TTTCCACGCCTACCACAGTC | AGAGGGGAAAAGTGCAAGCA | 250-300 | 54 |
| DCR SSR-13 | GAGGTTGATCCACAGCAGCT | GCATTGCAGGCACAAGAACA | 250-300 | 55 |
| DCR SSR-14 | GATCACACGCACGATGAAGC | CAAGCCCCCTTTTTCTGTGC | 230-300 | 56 |
| DCR SSR-15 | CGCGATGGGAATTCTACGGA | CCCACACAGCGATCTCAGTT | 260-300 | 54 |
| DCR SSR-16 | AATGCTCGCTCAAAGGGGAT | GGATTAGGAGGGTGGGACCT | 180 | 54 |
| DCR SSR-17 | GGTGGTATGTTGGAAGCCCA | TGGGAGCTAACCTAGAGCACA | 240-275 | 52 |
| DCR SSR-18 | GTTCTATTTTGGGCGGCCAC | GACAAAGTCCCACCCGCATA | 150-200 | 54 |
| DCR SSR-19 | TGGCCAAGATGTCGAATCCC | ACACATACCTGTCCTTGGCC | 160 | 54 |
| DCR SSR-20 | ATCGCCACCTACCCAACAAG | GCAATGCAAAGTATGAGGGTGG | 250-275 | 52 |
| DCR SSR-21 | TGGATTTGAGAGGGTTCAAGGG | ACACACGAGTCTGTGTCCAA | 75-240 | 56 |
| DCR SSR-22 | CCGTGTGTGTGTGTCTGAGA | GCTGAGGCATCTCTTTGGGT | 150-250 | 54 |
| DCR SSR-23 | AGTCATCGTCGCTGATTACG | ACCTGCTGTGTTGATCAGACT | 175-250 | 54 |
| DCR SSR-24 | TGAAGCGGAGAACCCTTTGG | AATCGTCCGTGATCAAGGCC | 245 | 53 |
| DCR SSR-25 | CATTAAGGCGGGCAGTCTCA | GTGGCTGGTTGTGTTTGCTT | NCA | 54 |
| DCR SSR-26 | CCTCAACCCAAGCAAGCAAC | AGCACAACAGGTACCTTCGG | 260 | 54 |
| DCR SSR-27 | GGGGCATCTGTGGGATTCAA | TGAAGGTACCTGCTGTGCTG | 120-200 | 54 |
| DCR SSR-28 | AGATGTGTGTGGGCTTCAGG | TCCGCACTCTTCAGCTTGTT | 250-300 | 54 |
| DCR SSR-29 | TGGTTGGATTTCCCCTTGGA | ATTAACTGAACGCGAGCCCA | 175-250 | 54 |
| DCR SSR-30 | ACACTTCCCATGAACAAGGACT | CATTAGCTCGAGGCCTGACA | 240-300 | 54 |
| DCR SSR-31 | AGTTTGAGGCGGGGACAAAA | TCTATTATGGGCGGCCACAC | NCA | 54 |
| DCR SSR-32 | CTGGCTAACGGGAGGTTTGT | GAGAGAGGGAGAAGGGGAGG | 245-290 | 54 |
| DCR SSR-33 | ACCAATCCCACCAGCAACAA | GCACAGACACACACACACAC | 200-250 | 54 |
| DCR SSR-34 | CTCTACCCACTCACCGAGGA | AGCACGTTCCACAAGGTTCA | 220-275 | 54 |
| DCR SSR-35 | TGGAGGTGTTTGGGATGCAA | TCCCCATTTGTGGTTGTGCA | 250-300 | 54 |
| DCR SSR-36 | GTCTGATCAGCACAGCAGGT | CCCTCAAAACCCAAGCAAGC | 250-275 | 54 |
| DCR SSR-37 | GGGTGGGGTGAGTTTCCATT | GGCCAACCCAGCTTGAAATG | 230-250 | 54 |
| DCR SSR-38 | GGCAACACGTCACCTGGATA | TTAAGAATGCCTGGGCCACA | 200-250 | 54 |
| DCR SSR-39 | AATAAGAGCACCTCGGCAGC | GCACGTTGCACATGTTTTCG | 250-275 | 54 |
| DCR SSR-40 | AAAAGGTGGAGCTGATGGCA | ACCCAACACATTAAGGTGCCA | 180 | 54 |
| DCR SSR-41 | TGTCTACACCTGTTTCTCCGT | GGCAAGTAGTAGCTCCACCC | 250-300 | 56 |
| DCR SSR-42 | CAAGAGGCCCCAAAAACAGC | GCCTGCCACCCTCACAATAT | 250-325 | 54 |
| DCR SSR-43 | TGTCCAGGGAGAGTGACTGT | TTTCAGCTGGCATGCCCATA | 250-300 | 52 |
| DCR SSR-44 | TTTTGGGTTGGCAATGGCTG | CTCATCGAGGTTGGTTGCCT | 225-250 | 54 |
| DCR SSR-45 | CCCCTGCAATTTTCCACGTG | AAACCCTCAGAGCTGATGCC | 250-275 | 54 |
| DCR SSR-46 | GTTGGAACAGGCGACCTACA | ACCATTTCGAACTGGAGCCA | 190-250 | 54 |
| DCR SSR-47 | ACCCACAGCTAGCCCAAATC | GGCAAGCCTAGGCCTACAAA | 225-275 | 54 |
| DCR SSR-48 | GCGGGAAAACATGTGGTGTG | CATGTTGTGGCTTGCATGCT | 150 | 54 |
| DCR SSR-49 | TGTGCGTGTTTTGACCCAAC | CCATGTGATGGGACCGGATT | 200-225 | 54 |
| DCR SSR-50 | GTGGGACTTTGTCGTTTGGC | ACTAAGGAGCACGACCATGC | 200 | 54 |
| DCR SSR-51 | GGGGTGAGTAGTTGGCCATC | AGGAGCTGTACGCAAAACCA | 225-250 | 54 |
| DCR SSR-52 | GCTTGAGTTTGGCTTGGCTT | TCCACACAAGGCCACAAGTT | 200-225 | 54 |
| DCR SSR-53 | TGGAGTTACCCACCTGTACCA | GGCTGTGAAGAAGTGTTTCGC | 225-275 | 54 |
| DCR SSR-54 | TCGTAACCTCCCTTGAGCAAC | ACATGAGCGGTCACAAGCTT | 200-225 | 54 |
| DCR SSR-55 | GGAATTCCAAAGGGCCTACCA | AGGATGGTGGGAGAGCATTA | 225 | 54 |
| DCR SSR-56 | GGCTTGAGTGCTGAATCCCT | TCATAAACTTGCCCTTGGGCA | 225-250 | 54 |
| DCR SSR-57 | CGCGCTCCTCCTGATTATGT | TCTCCACCCACTCGTACGAT | 185 | 54 |
| DCR SSR-58 | TGCTCCTGCCTTTGTGCTAA | ACACGAAAACTTCAACGTGGG | 200-225 | 54 |
| DCR SSR-59 | ACCCAGTTTAACAGGCTGAA | TTTTCCTCCGCTTCTCGCAT | 200-250 | 54 |
| DCR SSR-60 | GGGAATGGTGGGGGTAAAGG | TCTCATCAGAGCCACCTCCA | 225-250 | 55 |
| DCR SSR-61 | GATCACGTCGCATTTGCCTC | CCTTAACCGGTTGGGCATCT | 180 | 54 |
| DCR SSR-62 | TCCTTGCTGGCGACTTTGAT | TTCGTCCCCTTCCTCTTCCT | 175-200 | 54 |
| DCR SSR-63 | TGGATTCTCCCTTCCCCCTT | AATAGGCCAGGGAGGTGAGA | 225-250 | 54 |
| DCR SSR-64 | AGCACAACAGGTACCTTCGG | CCCTCAAAACCCAAGCAAGC | 200 | 54 |
| DCR SSR-65 | CAAGGGCACACATACCTGGT | TGATCAGCACAGCAGGTACC | 180 | 54 |
| DCR SSR-66 | GTGCGCGTCAACTTTCCTTT | TCTCCTCCGCCTCTGTTCTT | 220 | 54 |
| DCR SSR-67 | TGATAGGCCGAATCAGCGAC | ACTATTGTTCTCTGGCGGCC | 200-225 | 54 |
| DCR SSR-68 | GGCAGTGTCTGGTAGGTCAC | TTGATCAAGAGGCCAGTCCG | 225-250 | 54 |
| DCR SSR-69 | CAAGCAGCAAACAAGGGCAT | AACACAGCAGGTACCTTCGG | 200-250 | 54 |
| DCR SSR-70 | CGGTAGGTTTTAGGGCTCCG | AACTCATGGGGCACTGTCTG | 200-250 | 56 |
| DCR SSR-71 | GGAAATCCGGGAAAGGGTCA | GGCCAGCCAACCTAATGACT | 200-225 | 56 |
| DCR SSR-72 | GTTTGGTAGAGGGATCCTGCA | GAAGGTGTTACGGTGGCTCA | 175-200 | 56 |
| DCR SSR-73 | TGGAGAAGGAGGAGGACGAG | CTCCTTCTCCTCCTCGTCCA | 210-240 | 56 |
| DCR SSR-74 | CCCTACCAGAAGCCGAATGG | CATCCATAGCTCTTCGGCGT | 190-220 | 56 |
| DCR SSR-75 | TGCTGCTTCAAACCAATGTCG | AGCTTGGTGAACTCGAGCTT | 170-200 | 56 |
| DCR SSR-76 | TCGCCTTTCTGTCACAGCTT | AGGTCTTGGCATGAACCACA | 190-220 | 56 |
| DCR SSR-77 | AATTATTGCGGGCCTTGTGG | GAGAATTCACTCGGCAGGAGA | 100-130 | 56 |
| DCR SSR-78 | CGGAATGCAGCCAAAAACCA | ATGGTGGAATTCCGAACCCC | 240-260 | 56 |
| DCR SSR-79 | ACCGTCCCAACTCAATCTCA | TTGAGGAAACCTTGGGTGGG | 185-210 | 56 |
| DCR SSR-80 | AATCAGTTCCTCTCTGCGGC | ATTCTCTGAGCTTCCAGGGC | 275-290 | 56 |
| DCR SSR-81 | GGGAACTGCTGGGTGAGAAA | GAGCTGTGTGGCCTTCGTAT | 250-275 | 54 |
| DCR SSR-82 | AAGCAAGCAGGATCAGACCC | CACACACACACACACACACA | NCA | 56 |
| DCR SSR-83 | GGCTTTTTCTTTGGGCAGGG | GTCTTCTTCACCGACCCCTG | 220 | 53 |
| DCR SSR-84 | AGGTACATGTGGGCAGTTGG | GTAGCAACTAGGCAGCCCAA | NCA | 54 |
| DCR SSR-85 | AGGGATTTCTTGTTTGGCGT | ACGGGTAAGTTCTTGGTTCCA | 175-200 | 56 |
| DCR SSR-86 | TGGCAATGAGTAGCAGCACA | TCAACTGAGGCAAGCTCACC | 180 | 56 |
| DCR SSR-87 | CAGCTTCCCAGACCCCAAAT | ATCAGTGGCTTGTGTAGGGC | 190 | 56 |
| DCR SSR-88 | ACAACAACACCCACCACCAT | TTGCCAGTTCTTGCATGCAC | 230-245 | 56 |
| DCR SSR-89 | AAAAGCTTGAGTGCATGCAC | GTGCCCTAGCTCCATACATGT | NCA | 56 |
| DCR SSR-90 | GGCAATTGGAGTGCTCATGC | GGCTTACTTCCTTGCGATGG | 150 | 55 |
| DCR SSR-91 | TTCACCCATGAGTGTGTGCA | GAATTTGTTGGGCTTGGTCCA | NCA | 56 |
| DCR SSR-92 | TGTGGGGGTGTGTTTGGAAA | CAGACAGCAGGTGACCCATT | 125 | 56 |
| DCR SSR-93 | GGTGGGTTGGGACAGAAGTT | GTGCACAAAGGTCCACAAACA | 220 | 56 |
| DCR SSR-94 | AGGGTTTTTGAGTTGTGCGT | ATCAGGAGTAAGGCGCATGG | 250 | 54 |
| DCR SSR-95 | TGAAGGGCCCATTTCTCCAC | CCCCGTGATCAGGATAGCAC | 150 | 56 |
| DCR SSR-96 | TCACTTGGAGGCTTGTTTCTCT | ACGTGAGGTAATGGAAGATGGT | 150 | 55 |
| DCR SSR-97 | TCCACACACACACACACACA | CTTTGGGATGACACTCCCCA | NCA | 56 |
| DCR SSR-98 | TGTGCGCAATGGGAAAGTTG | CGACACATGGAACCCCTTGA | 200 | 56 |
| DCR SSR-99 | ACAATCCAAAGCATGCGAGC | CTTGCAGGTTGGTGTTTCGG | 175 | 55 |
| DCR SSR-100 | CTCTCTCGGCTCTTGTCGTC | AAGCGAGGAGAATGCGAGAG | 150-200 | 56 |

*NCA=Not Cross-species Transferable
